# A scoping review of factors potentially linked with antimicrobial-resistant bacteria from turkeys (iAM.AMR Project)

**DOI:** 10.1017/S0950268822001224

**Published:** 2022-07-18

**Authors:** Charly Phillips, Brennan Chapman, Agnes Agunos, Carolee A. Carson, E. Jane Parmley, Richard J. Reid-Smith, Ben A. Smith, Colleen P. Murphy

**Affiliations:** 1 Public Health Risk Sciences Division, National Microbiology Laboratory, Public Health Agency of Canada, Guelph, ON, Canada; 2 Department of Population Medicine, Ontario Veterinary College, University of Guelph, Guelph, ON, Canada; 3 Foodborne Disease and Antimicrobial Resistance Surveillance Division, Centre for Food-borne, Environmental and Zoonotic Infectious Diseases, Public Health Agency of Canada, Guelph, ON, Canada

**Keywords:** Antibiotic resistance, antimicrobial resistance in agricultural settings, Canada, foodborne infections, turkeys

## Abstract

Antimicrobial resistance (AMR) is a complex problem that is a threat to global public health. Consumption of turkey meat may be an important source of foodborne exposure to resistant bacteria; recent outbreaks of multi-drug-resistant *Salmonella* Reading in Canada and the USA have implicated raw turkey products. To better understand the epidemiology of AMR in farmed turkey production, a scoping review was conducted. The objectives were to identify (1) modifiable factors potentially associated with antimicrobial-resistant *Campylobacter*, *Enterococcus*, *Escherichia coli* and *Salmonella enterica* along the farm-to-fork pathway in turkeys, and (2) data gaps with respect to factors potentially associated with AMR and Canadian commercial turkey production. A comprehensive search of the peer-reviewed literature was conducted in 2019 and updated in 2021. Thirteen references were included, reporting 36 factors. Antimicrobial use factors and their potential associations with AMR were most frequently reported (*n* = 15 factors; 42%), followed by biosecurity (*n* = 11; 31%) and management practices (*n* = 10; 28%). This review revealed important data gaps; no factors pertaining to *S. enterica* or to stages other than the farm (e.g. abattoir, retail) were identified, and only one Canadian reference was identified. These findings will inform priorities for future research and surveillance regarding turkeys and AMR.

## Introduction

Antimicrobial resistance (AMR) is a current global threat to public health [1]. Losses of antimicrobial treatments jeopardise modern medicine, including routine procedures like cancer treatment [2]. Resistant infections pose an increased risk of death compared to non-resistant infections, and they may contribute to other components of burden of illness, such as lengthened hospital stays [2, 3]. Antimicrobial use (AMU) in food-producing animals has been associated with AMR in the farm-to-fork pathway (farm, abattoir, retail), as well as resistant infections in humans [4, 5]. For example, ceftiofur use in broiler chicken hatcheries has been related to ceftiofur resistance in clinical *Salmonella* Heidelberg human isolates [5]. Transmission of resistant bacteria or mobile genetic elements carrying resistance determinants occurs readily, both within and between human, animal and environmental reservoirs [4]. Humans can be exposed to AMR from the farm-to-fork pathway through direct animal contact, ingestion of contaminated foods of animal origin or crops, or contact with the environment [6]. Addressing AMR linked with agriculture, and – in particular – food-producing animals is a key priority in Canada [7].

Farmed turkeys (referred to as turkeys herein) are an important food animal species in Canada, encompassing commercial turkeys, which are slaughtered for meat, and breeder turkeys, which supply hatching eggs destined for commercial turkey production. Turkey meat is the fourth most commonly consumed meat (following chicken, beef and pork) in Canada, and ranks second for consumption as deli meat [8]. Furthermore, turkey is a staple of the Thanksgiving and Christmas holidays; over 70% of annual whole turkey purchases in Canada are associated with these holidays [9]. Foodborne outbreaks have been linked with turkey meat consumption in Canada and the USA, particularly outbreaks of *Salmonella enterica* serovars Reading [10, 11], Schwarzengrund [12], Heidelberg [13, 14] and Hadar [15], some of which spanned multiple jurisdictions and were multi-drug-resistant (MDR) [10, 11, 14, 15]. Notably, two outbreaks of MDR *S.* Reading linked to raw turkey products occurred between 2017 and 2020 in Canada, and between 2017 and 2019 in the USA [10, 11].

Although it is well-established that AMU creates conditions for the emergence, dissemination and persistence of AMR, other factors may influence AMR in the farm-to-fork pathway, such as housing conditions at the farm, or carcass chilling during meat processing [4, 16]. Overall, the relative importance and combined effect of the multiple factors along the farm-to-fork pathway that influence the emergence of antimicrobial-resistant infections in humans is poorly understood [16, 17]. Moreover, there is a large volume and variety of published AMR literature encompassing multiple antimicrobials, bacteria, interventions, metrics, sampling methods, study designs and timeframes [16]. Hence, there is a need for synthesis of this information. Synthesis research methodologies are structured, transparent and reproducible approaches for facilitating evidence-based policy-making [18]. Their suitability for policy questions in agri-food has been widely endorsed [19]. Scoping reviews are suited to address broad questions, and involve identifying, charting and summarising the literature within a particular topic, as well as highlighting data gaps [18].

Existing knowledge synthesis literature on factors potentially linked with AMR in livestock has focused on cattle, chickens and pigs [16, 20, 21]. The Canadian Integrated Program for Antimicrobial Resistance Surveillance (CIPARS) reported that over half of turkey-origin *Campylobacter*, *Escherichia coli* and *S*. *enterica* on-farm isolates were resistant to at least one antimicrobial class [22]. This included resistance to antimicrobials classified as ‘Critically Important’ or of ‘Very High Importance’ to human medicine by the World Health Organization (WHO) and Health Canada, respectively [22–24]. Review articles that have included turkeys are often limited to analyses of AMR prevalence [25, 26] or have reported findings where turkeys are aggregated with other poultry species [27]. Synthesis research addressing factors potentially linked with AMR in turkeys separately from other food animal species has either targeted one bacterial species [28] or utilised a narrative review methodology [29]. CIPARS has reported differences between broiler chickens and turkeys, including differences in AMR profiles, recovery of foodborne bacteria, distribution of *Campylobacter* species and composition of *Salmonella* serotypes [22]. As well, operational practices (e.g. sources of hatching eggs and turkey poults, marketing weights and industry-driven AMU stewardship activities, proportion of conventional *vs.* organic or raised without antibiotics flocks) and animal health parameters (e.g. vaccination, diseases) vary substantially between poultry species raised in Canada [30–34]. On account of these differences, data about broiler chickens cannot be extrapolated to turkeys. Thus, factors potentially linked with AMR in turkeys warrant specific consideration.

Given apparent gaps in the AMR literature regarding turkeys, a scoping review was conducted. The objectives were: (1) to qualitatively describe the available peer-reviewed literature reporting modifiable factors potentially associated with the occurrence of antimicrobial-resistant *Campylobacter* species, *Enterococcus* species, *E. coli* and *S. enterica* along the farm-to-fork pathway in turkeys, and (2) to describe data gaps in this literature with a particular focus on Canadian commercial turkey production. A factor was defined as a measured observation with an investigated potential association or relationship with AMR, such as AMU at the farm or the use of disinfectants at the abattoir [16].

This scoping review was conducted as part of a larger project: the Integrated Assessment Modelling of Antimicrobial Resistance (iAM.AMR) project [16]. The goal of the iAM.AMR project is to quantitatively model AMR along the farm-to-fork pathway for the main food animal species in Canada. Initial steps for many components of the iAM.AMR project are scoping reviews to identify and describe modifiable factors potentially associated with AMR in the main food animal species in Canada. Results of the scoping review for turkeys are presented herein (findings from the other components of the iAM.AMR project will be published separately). Since the primary focus of our research group is AMR in Canada, and regional variation in agricultural practices impacts the factors investigated in primary research [16, 35], this review emphasised factors that are from Canadian studies or have relevancy to Canadian turkey production.

## Methods

### Literature search

This scoping review adheres to the PRISMA-ScR (Preferred Reporting Items for Systematic reviews and Meta-Analyses extension for Scoping Reviews) guidelines for the reporting of scoping reviews [36]. A protocol was not registered.

The research team had expertise in AMR, food safety, epidemiology, veterinary medicine, poultry, library science and synthesis research methodologies. As the initial search was designed to capture data for all facets of the iAM.AMR project (i.e. other food animal species in addition to turkeys), the search was broader than necessary for this review, yet appropriate to address the specific objectives of this review.

The initial search was run on 11 April 2019 in five databases: Medline, Embase, Agricola, Centre for Agriculture and Biosciences International Abstracts (CAB Abstracts) and Food Science and Technology Abstracts (Supplementary Tables S1, S3–S6). For this review, the search was updated on 11 August 2021, where the host species was limited to turkeys (Supplementary Tables S2, S7–S10). All Supplementary material is available online on the Cambridge Core website.

All citations retrieved were imported into reference management software (RefWorks 2.0; ProQuest LLC, Bethesda, Maryland, USA), where exact matches were de-duplicated automatically. Citations were then imported into the web-based systematic review software Rayyan [37]. All potential duplicates identified by Rayyan's duplicate filter were reviewed by a single reviewer and verified duplicates were removed manually. The results from the updated search were de-duplicated against the results from the initial search.

### Eligibility criteria

**Fig. 1.**
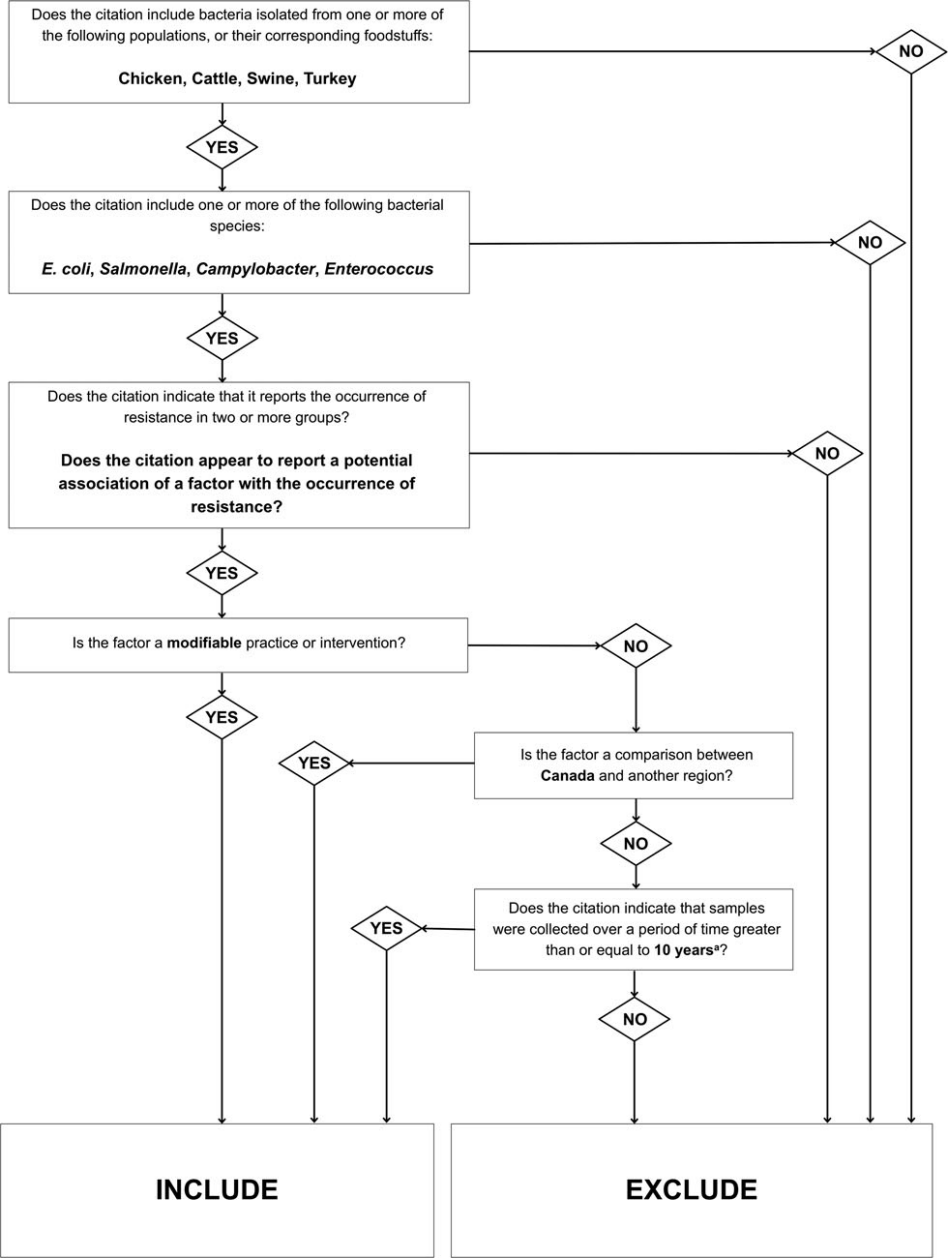
Flowchart depicting the criteria used to guide reviewer decisions at primary (title/abstract) screening. ^a^Citations were excluded if the year of sampling was the only factor reported, as year was deemed a non-modifiable factor. However, if the abstract indicated that samples were taken over a time period greater than or equal to 10 years (and the citation passed all previous primary screening questions in this flowchart), the citation was included, as this time period was potentially extensive enough to include samples taken before and after AMU policy changes, which are modifiable factors. If such policy changes were described in the full text (at secondary screening), the reference was potentially eligible for inclusion at secondary screening.

**Fig. 2.**
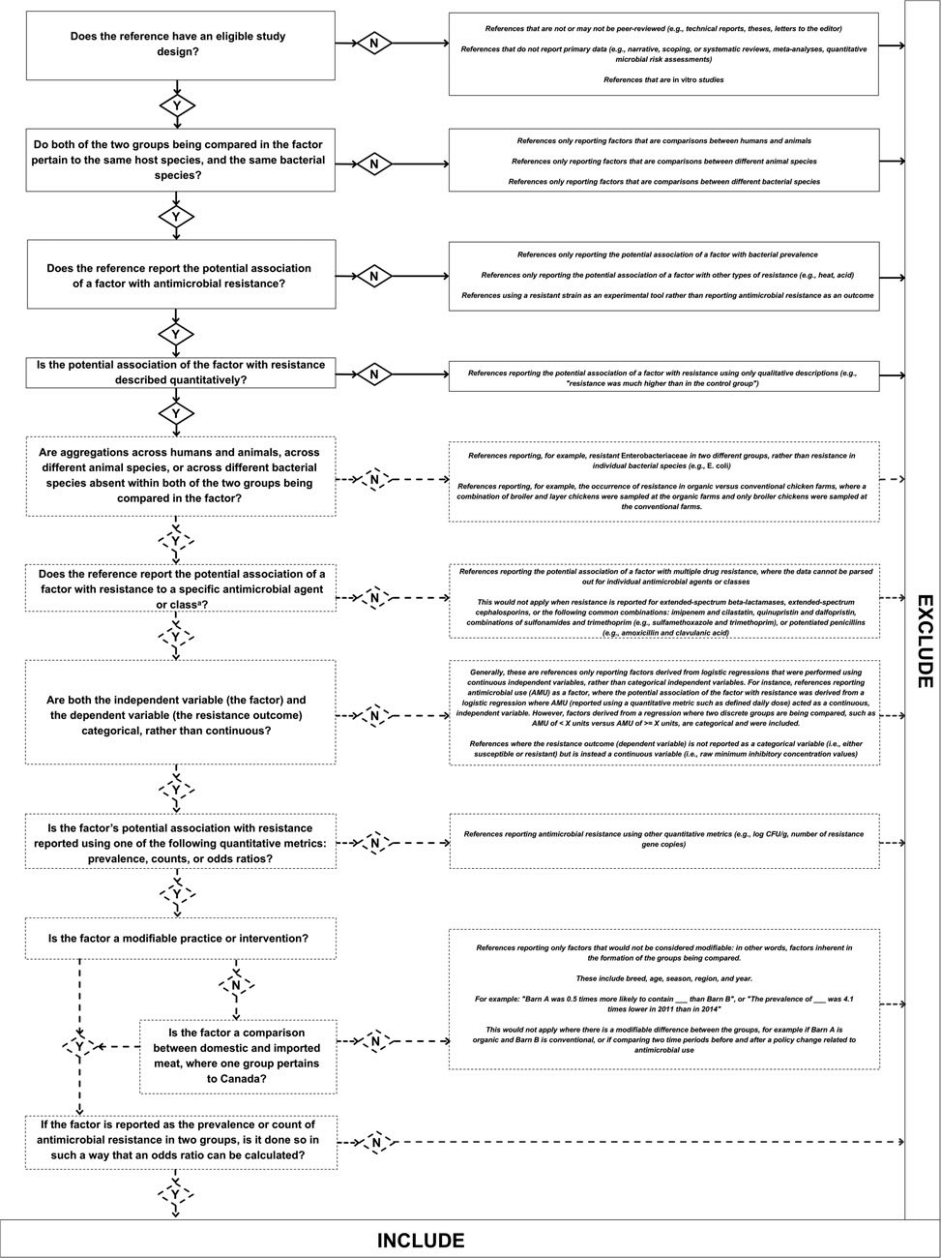
Flowchart depicting the criteria used to guide reviewer decisions at secondary (full-text) screening. Y, yes; N, no. Exclusion decisions displayed here were sometimes made at the data extraction stage, rather than at secondary screening, as the applicability of these reasons to particular references were more obvious in some cases than in others. All shapes with dotted lines represent those exclusion decisions that more often required the extra scrutiny applied at the data extraction stage, while shapes with solid lines represent exclusion decisions that were made at the secondary screening stage. ^a^This includes references reporting a potential association between a factor and the occurrence of an AMR gene, as long as the potential association was reported in a specified, relevant bacterial species.

The eligibility criteria are outlined in Figures 1 and 2. Peer-reviewed publications written in the English language from any geographical region and date of publication were eligible (Fig. 2). *In vitro* studies, reviews and other synthesis methodologies (e.g. meta-analyses) were excluded. Analytic studies were eligible, including observational and experimental studies.

References reporting a modifiable factor potentially linked with the occurrence of resistance were included if resistance was reported quantitatively in such a way that an odds ratio could be calculated (to account for the needs of the larger iAM.AMR project) [38] (Figs 1 and 2). Potential associations between factors and AMR eligible for data extraction were not limited to those interpreted as statistically significant (in alignment with previous synthesis work conducted as part of the iAM.AMR project) [16]. For the purposes of this review, a factor was extracted if AMR was reported in two groups: (1) the factor group (a study group in which the factor was present or applied, e.g. a group in which ceftiofur was administered); and (2) the comparator group (a study group in which the factor was not present or applied, e.g. a group in which ceftiofur was not administered), or if an odds ratio describing a potential association between these groups and AMR was reported. Eligible references did not need to report statistical analyses between factors and AMR or interpretations of statistical significance.

References only reporting modifiable factors potentially linked with the occurrence of multiple drug resistance in such a way that resistance to individual antimicrobials or antimicrobial classes could not be parsed out were excluded (Fig. 2). However, exceptions were made for resistance to extended-spectrum beta-lactamases, extended-spectrum cephalosporins and the following common combinations: imipenem and cilastatin; quinupristin and dalfopristin; combinations of sulphonamides and trimethoprim and potentiated penicillins.

Furthermore, references only reporting factors analysed as continuous variables, rather than categorical variables, were excluded given the objectives for this stage of the overall model development for the larger iAM.AMR project (Fig. 2). The eligibility criteria for this scoping review were informed by the requirements for the iAM.AMR project's current modelling objectives, which include: (1) each factor must have a discrete comparator group, in order to understand the potential links between factors and the occurrence of AMR; and (2) each factor must be assigned a frequency of occurrence (e.g. the frequency of farms that reported the use of ceftiofur, for a factor comparing ceftiofur use and no ceftiofur use) [38]. Categorical measures of AMU have been useful for assessing the impact of an AMU intervention strategy on AMR [34] and restricting initial stages of model development to factors analysed as categorical variables is a pragmatic choice for the iAM.AMR project. Thus, for this stage of the larger iAM.AMR project, assigning eligible factors to categorical groups (i.e. the factor and comparator groups) was necessary and restricted eligibility for this review to this type of reporting.

### Relevance screening and data extraction

Primary (title/abstract) screening was performed in Rayyan, and secondary (full-text) screening was performed in Excel (Excel 2016, Microsoft Corp., Redmond, Washington, USA), using the criteria given in Figures 1 and 2.

At each stage, citations or references were screened independently by two reviewers. Conflicts were resolved by consensus and where consensus could not be reached, citations or references were screened by a third reviewer. If an abstract was not available at primary screening, the citation was included and the full text was sought in preparation for secondary screening. At secondary screening in the initial search, selections of ‘unsure’ were either (1) resolved as conflicts, where references were included if both reviewers were unsure, or (2) resolved by a third reviewer, where references were included if the third reviewer was unsure. References were omitted from secondary screening if: the full text was not available, the reference was published in a language other than English, the reference was provided in an alternate format (e.g. conference abstract) or the reference was not unique (e.g. a duplicate of another returned reference, or a dissertation later published in a peer-reviewed reference captured by the search).

References included at secondary screening were imported from Excel into a custom data extraction tool (Access 2016, Microsoft Corp., Redmond, Washington, USA). Characteristics of the references, factors and potential associations with resistance were entered into the data extraction form provided in this database (Supplementary Table S11). Each reference was extracted by a single extractor.

### Validation and synthesis of results

Because the initial search included other species in addition to turkeys, it was necessary to identify all references pertaining to turkeys (hereafter referred to as turkey references). Bibliographic data for all references included at secondary screening from the initial search were imported as an Excel file into R (R Foundation for Statistical Computing, Vienna, Austria), where a custom script was used to identify potential turkey references based on the presence of at least one of the following terms: ‘turkey’, ‘poult’, ‘poultry’, ‘broiler’, ‘avian’, ‘flock’, ‘Meleagris’, ‘fowl’, ‘hen’, ‘tom’ or ‘gobble’, in the title or abstract fields. Potential turkey references identified by this script that also reported sampling turkey-origin bacteria in the full text were retained for this review. The data extraction results for these retained turkey references from the initial search, in addition to the data extraction results from the updated search (all of which pertained to turkeys), were then validated against the full text and corrected, if necessary, by a single reviewer, with input from a second reviewer. Exclusion criteria for data extraction of turkey references were the same as the secondary screening criteria (Fig. 2).

The reviewed and corrected data extraction results were then exported from the Access database into Excel. Factors were evaluated for common themes. Themes were not identified *a priori*. Descriptive statistics (counts and frequencies) were calculated for factor-level characteristics (details of the study groups and populations in which a factor was present or absent) and outcome-level characteristics (the bacterial species and antimicrobial classes for which resistance was reported) in Excel. For factor-level counts, a factor was deemed a unique combination of the: (1) factor group, (2) comparator group and (3) turkey sub-population (breeder or commercial). If AMU factors involved the administration of the same antimicrobial or antimicrobial class, they were aggregated together for factor-level counts, even if they involved different doses or routes of administration.

## Results

### Selection of sources of evidence

**Fig. 3.**
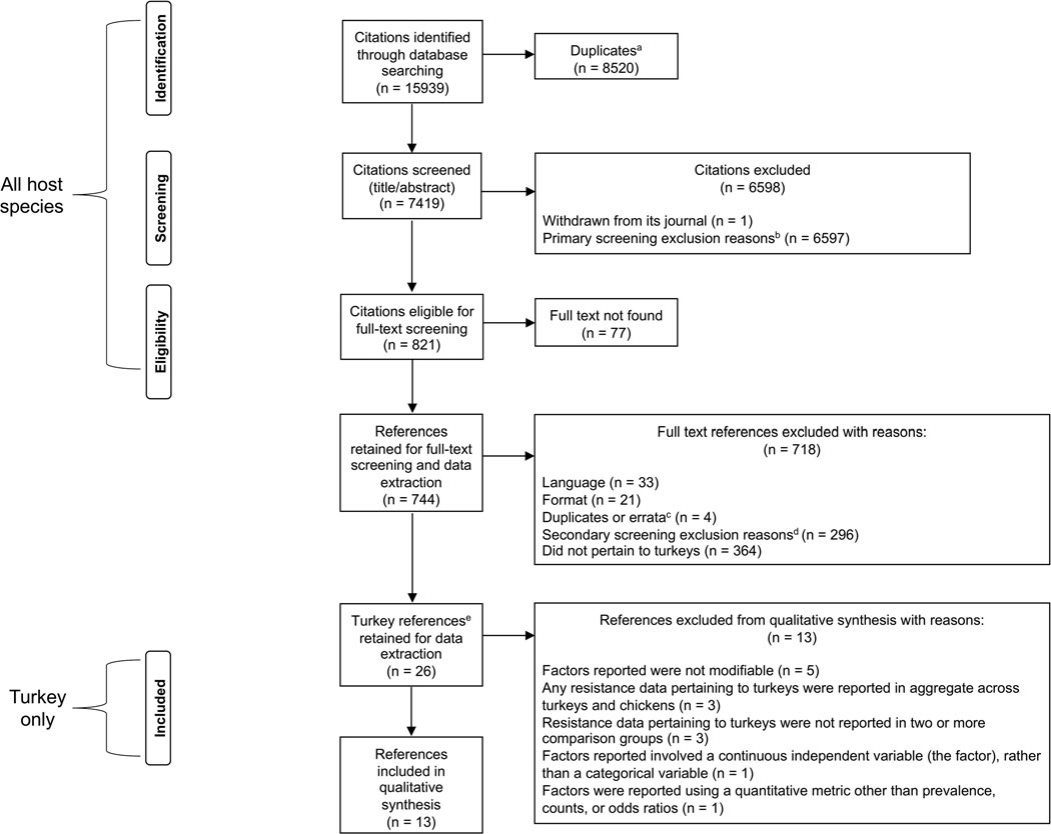
PRISMA flow diagram of citations and references through the scoping review process to identify factors potentially associated with AMR in *Campylobacter* species, *Enterococcus* species, *E. coli* and *S. enterica* from turkeys. Counts at each step in the flow diagram reflect the totals for the initial and updated searches combined. ^a^These include: (1) duplicates within the returns of each search, and (2) duplicates that emerged when the results of the updated search were de-duplicated against the results of the initial search. ^b^A detailed description of the exclusion criteria at primary (title/abstract) screening is available in Figure 1. ^c^Errata were not counted as individual references starting at full-text screening, as they were paired with their corresponding full-text articles. ^d^A detailed description of the exclusion criteria at secondary (full-text) screening is available in Figure 2. ^e^Turkey references: references which reported sampling turkey-origin bacteria in the full text.

The initial and updated searches returned a combined total of 7419 unique citations for primary screening (Fig. 3). Of these, 744 were retained for secondary screening. Following secondary screening, 26 references pertained to turkeys, and 13 were relevant to this scoping review and underwent qualitative synthesis (Fig. 3). Of the 13 included references, 10 were captured in the initial search and three were newly identified in the updated search.

### General characteristics of sources of evidence

Characteristics of all identified sources of evidence are presented in Tables 1–4. Of the 13 references included, six were from the USA, two each were from France and Germany and one each was from Canada, Great Britain and Italy [39–51]. Six references were published between 2016 and 2020, six were published between 2005 and 2016 and one was published in 1982. All but three references involved observational studies.

**Table 1.** Description of factors (AMU: AMU policy change and binary qualitative AMU themes) potentially associated with AMR in *Campylobacter* species, *Enterococcus* species and *E. coli* from turkeys

Population	Study design	Factor group^a^	Comparator group^b^	Bacteria	Resistance outcome	Location	Reference
Commercial turkeys	Observational	After French Act No. 2014-1170 went into effect, which imposed incentive tools to reduce the use of critically important antimicrobials^c^ (e.g. third and fourth-generation cephalosporins and fluoroquinolones), such as prohibitions on discounts, rebates and reductions (sampling from 2016)	Before French Act No. 2014-1170 went into effect (sampling from 2014)	*C. jejuni*	Ciprofloxacin	France	[39]
					Erythromycin		
					Gentamicin		
				*E. coli*	Ciprofloxacin		
					Nalidixic acid		
		After both Decree 2016-317, which banned the preventive use of critically important antimicrobials^c^, and French Act No. 2014-1170 went into effect (sampling from 2018)	Before the passage of either French Act No. 2014-1170 or Decree 2016-317 (sampling from 2014)	*C. jejuni*	Ciprofloxacin		
					Erythromycin		
					Gentamicin		
				*E. coli*	Ciprofloxacin		
					Nalidixic acid		
		Ceftiofur use (in hatchery)	No ceftiofur use (in hatchery)	*E. coli*	Ceftiofur	Canada	[47]
		Enrofloxacin use in the last year	No enrofloxacin use in the last year	*E. faecalis*	Ciprofloxacin	USA	[48]
				*E. faecium*			
		Gentamicin use (in hatchery)	No gentamicin use (in hatchery)	*Enterococcus* spp.	Gentamicin	Canada	[47]
				*E. coli*			
		Tylosin use (preventive in-feed)	No tylosin use (preventive in-feed)	*Enterococcus* spp.	Tylosin		
		Virginiamycin use (preventive in-feed)	No virginiamycin use (preventive in-feed)		Quinupristin and dalfopristin		
		Virginiamycin use in the last year	No virginiamycin use in the last year	*E. faecium*	Quinupristin and dalfopristin	USA	[48]
		Bacitracin use (preventive in-feed)	No bacitracin use (preventive in-feed)	*Enterococcus* spp.	Bacitracin	Canada	[47]
		Tetracycline use (preventive in-feed or as treatment)	No tetracycline use (preventive in-feed or as treatment)	*E. coli*	Tetracycline		
		Any antimicrobial treatment administered to the flock	No antimicrobial treatment administered to the flock	*E. coli*	Ciprofloxacin	Great Britain	[50]
		Any subtherapeutic AMU (in-feed)	No in-feed AMU for the last 3 or more years	*E. coli*	Gentamicin	USA	[45]
					Neomycin		
					Sulphathiazole and trimethoprim		
					Clindamycin		
					Erythromycin		
					Tilmicosin		
					Ampicillin		
					Penicillin		
					Spectinomycin		
					Florfenicol		
					Sulphadimethoxine		
					Chlortetracycline		
					Oxytetracycline		
					Tiamulin		
		Subtherapeutic AMU (in-feed)	No AMU via feed or water^d^	*E. coli*	Gentamicin	USA	[41]
Breeder turkeys	Observational	Fluoroquinolone treatment in the last year	No fluoroquinolone treatment in the last year	*E. coli*	Ciprofloxacin	Great Britain	[50]

a Factor group: the study group in which the factor was present or applied (e.g. a group of turkeys in which chlortetracycline was administered in-feed).

b Comparator group: the study group in which the factor was not present or applied (e.g. a group of turkeys in which chlortetracycline was not administered in-feed).

c As designated by the World Health Organization [23].

d Birds in both the factor and comparator groups were administered antimicrobials *in ovo* and as poults (eggs were dipped into a solution of tylosin (300 μl/ml) and gentamicin (500 ppm), and poults received 1 mg of gentamicin via injection after hatching) [41].

**Table 2.** Description of the factors (AMU: binary quantitative AMU theme) potentially associated with AMR in *Campylobacter* species, *Enterococcus* species and *E. coli* from turkeys

Population	Study design	Factor group^a^	Comparator group^b^	Bacteria	Resistance outcome	Location	Reference
Commercial turkeys	Observational	Neo-terramycin use (oxytetracycline hydrochloride at 106 mg/l and neomycin sulphate at 74 mg/l of H_2_O) for 3 days	No AMU via feed or water^c^	*E. coli*	Gentamicin	USA	[41]
		Chlortetracycline use (106 mg/l H_2_O) for 9 h	No AMU via feed or water^c^				
		Chlortetracycline use (106 mg/l H_2_O) for 3 days	No AMU via feed or water^c^				
		Chlortetracycline use (106 mg/l H_2_O) for 4 days	No AMU via feed or water^c^				
		Continuous chlortetracycline use (200 mg/kg), administered in-feed	No AMU via feed or water^c^				
		Nitrofurazone use (220 mg/kg) for 1 week, administered in-feed	No AMU via feed or water^c^				
		Nitrofurazone use (220 mg/kg) for 1 week and continuous chlortetracycline use (220 mg/kg), both administered in-feed	No AMU via feed or water^c^				
	Non-observational	Enrofloxacin use (10 mg enrofloxacin/kg body weight per day, via drinking water) for 5 days	No enrofloxacin use^d^	*E. coli*	Enrofloxacin	USA	[51]
					Ampicillin		
		Pulsed tylosin use (60 mg tylosin/kg body weight, via a therapeutic dose of 0.53 g/l H_2_O) in 3 separate dosing periods lasting 3 days each	No AMU (in water)	*Campylobacter* spp.	Erythromycin	USA	[42]
Breeder turkeys	Non-observational	Paromomycin sulphate use (100 mg/kg), administered in-feed from day of hatch to day 120	No paromomycin use	*E. faecium*	Teicoplanin	France	[49]
					Vancomycin		
					Gentamicin		
					Kanamycin		
					Streptomycin		
					Sulphamethoxazole and trimethoprim		
					Lincomycin		
					Erythromycin		
					Ampicillin		
					Pristinamycin		
					Chloramphenicol		
					Tetracycline		
				*E. coli*	Ciprofloxacin		
					Amikacin		
					Gentamicin		
					Kanamycin		
					Neomycin		
					Netilmicin		
					Paromomycin		
					Streptomycin		
					Tobramycin		
					Sulphamethoxazole and trimethoprim		
					Amoxicillin		
					Nalidixic acid		
					Chloramphenicol		
					Tetracycline		

a Factor group: the study group in which the factor was present or applied (e.g. a group of turkeys in which chlortetracycline was administered in-feed).

b Comparator group: the study group in which the factor was not present or applied (e.g. a group of turkeys in which chlortetracycline was not administered in-feed).

c Birds in both the factor and comparator groups were administered antimicrobials *in ovo* and as poults (eggs were dipped into a solution of tylosin (300 μl/ml) and gentamicin (500 ppm), and poults received 1 mg of gentamicin via injection after hatching) [41].

d The factor and comparator groups are representative of the same group of birds, sampled after and before antimicrobial administration, respectively [51].

**Table 3.** Description of factors (biosecurity theme) potentially associated with AMR in *E. coli*^a^ from turkeys

Population	Study design	Factor group^b^	Comparator group^c^	Bacteria	Resistance outcome	Location	Reference
Commercial turkeys	Observational	Close proximity of watercourse to poultry houses	Watercourse far from poultry houses	*E. coli*	Combination: ampicillin, plus ceftazidime and/or cefotaxime and potentially cefoxitin or ciprofloxacin as well^d^	Great Britain	[50]
		Designated gloves available for farm staff	Designated gloves not available for farm staff				
		Disinfection of floors and walls at depopulation	No disinfection of floors and walls at depopulation		Ciprofloxacin		
		Evidence of mice	No evidence of mice				
		Growth on litter used by chickens	Growth on un-used litter	*E. coli*	Ceftiofur	Canada	[47]
					Gentamicin		
		Pigs present on neighbouring farms	Pigs not present in neighbouring farms	*E. coli*	Combination: ampicillin, plus ceftazidime and/or cefotaxime and potentially cefoxitin or ciprofloxacin as well^d^	Great Britain	[50]
		Staff work with other livestock	Staff do not work with other livestock				
Breeder turkeys	Observational	Foot dips replenished more than once a week	Foot dips not replenished more than once a week	*E. coli*	Ciprofloxacin	Great Britain	[50]
		Horses present on neighbouring farms	Horses not present on neighbouring farms				
		Other domestic animals present on the farm	Domestic animals not present on the farm		Combination: ampicillin, plus ceftazidime and/or cefotaxime and potentially cefoxitin or ciprofloxacin as well^d^		
		Water sourced from main supply	Water not sourced from main supply				

a No biosecurity factors potentially associated with AMR in *Campylobacter* species or *Enterococcus* species were identified.

b Factor group: the study group in which the factor was present or applied (e.g. a group of turkeys in which chlortetracycline was administered in-feed).

c Comparator group: the study group in which the factor was not present or applied (e.g. a group of turkeys in which chlortetracycline was not administered in-feed).

d This reference reported cephalosporin resistance [50]. During bacterial isolation, media was infused with either cefotaxime, cefoxitin, ciprofloxacin or no antimicrobial. Disc diffusion was then applied as the antimicrobial susceptibility testing method, and the results of this testing were not separated based on the isolation media used. Third-generation cephalosporin resistance was defined as ‘resistant to ampicillin, plus ceftazidime and/or cefotaxime in the disc diffusion test’ [50], but cephalosporin resistance was not defined. Since this resistance outcome represents a combination of antimicrobial classes classified under categories I and II of importance to human medicine [24], it has been placed after factors for which a potential association with fluoroquinolone (category I) [24] resistance was reported.

**Table 4.** Description of factors (management practices theme) potentially associated with AMR in *Campylobacter* species and *E. coli*^a^ from turkeys

Population	Study design	Factor group^b^	Comparator group^c^	Bacteria	Resistance outcome	Location	Reference
Commercial turkeys	Observational	Age of the youngest birds in the flock is ≥105 days	Age of the youngest birds in the flock is <105 days	*E. coli*	Ciprofloxacin	Great Britain	[50]
		Feed sourced from a national compounder	Feed not sourced from a national compounder				
		Independent farm	Not an independent farm		Combination: ampicillin, plus ceftazidime and/or cefotaxime and potentially cefoxitin or ciprofloxacin as well^d^		
		Organic production	Conventional production	*C. coli*	Ciprofloxacin	Germany	[40]
					Gentamicin		
					Streptomycin		
					Erythromycin		
					Nalidixic acid		
					Tetracycline		
				*C. jejuni*	Ciprofloxacin		
					Gentamicin		
					Streptomycin		
					Erythromycin		
					Nalidixic acid		
					Tetracycline		
				*Campylobacter* spp.	Ciprofloxacin	USA	[43]
					Norfloxacin		
					Gentamicin		
					Kanamycin		
					Clindamycin		
					Erythromycin		
					Ampicillin		
					Nalidixic acid		
					Tetracycline		
				*E. coli*	Ciprofloxacin	Italy	[44]
					Colistin		
					Cefotaxime		
					Ceftazidime		
					Gentamicin		
					Kanamycin		
					Streptomycin		
					Ampicillin		
					Nalidixic acid		
					Chloramphenicol		
					Florfenicol		
					Trimethoprim		
					Sulphamethoxazole		
					Tetracycline		
					Imipenem	USA	[46]
					Ciprofloxacin		
					Ampicillin-sulbactam		
					Ceftriaxone		
					Amikacin		
					Gentamicin		
					Cefoxitin		
					Sulphamethoxazole and trimethoprim		
					Cefazolin		
					Ampicillin		
					Nalidixic acid		
					Tetracycline		
		Housing with partitions between flocks	Housing without partitions between flocks	*E. coli*	Ciprofloxacin	Great Britain	[50]
					Combination: ampicillin, plus ceftazidime and/or cefotaxime and potentially cefoxitin or ciprofloxacin as well^d^		
		Raised without antibiotics production	Conventional production	*E. coli*	Imipenem	USA	[46]
					Ciprofloxacin		
					Ampicillin-sulbactam		
					Ceftriaxone		
					Amikacin		
					Gentamicin		
					Cefoxitin		
					Sulphamethoxazole and trimethoprim		
					Cefazolin		
					Ampicillin		
					Nalidixic acid		
					Tetracycline		
Commercial turkeys	Non-observational	Birds moved to new pens (of an identical housing type) partway through a production cycle	Birds remained in the same pen for the entirety of the production cycle	*E. coli*	Enrofloxacin	USA	[51]
					Ampicillin		
Breeder turkeys	Observational	Birds raised in the same house from placement to de-population	Birds not raised in the same house from placement to de-population	*E. coli*	Combination: ampicillin, plus ceftazidime and/or cefotaxime and potentially cefoxitin or ciprofloxacin as well^d^	Great Britain	[50]
		Independent farm	Not an independent farm				
		Number of birds on the farm is >10 000	Number of birds on the farm is <10 000		Ciprofloxacin		

a No management practice factors potentially associated with AMR in *Enterococcus* species were identified.

b Factor group: the study group in which the factor was present or applied (e.g. a group of turkeys in which chlortetracycline was administered in-feed).

c Comparator group: the study group in which the factor was not present or applied (e.g. a group of turkeys in which chlortetracycline was not administered in-feed).

d This reference reported cephalosporin resistance [50]. During bacterial isolation, media was infused with either cefotaxime, cefoxitin, ciprofloxacin or no antimicrobial. Disc diffusion was then applied as the antimicrobial susceptibility testing method, and the results of this testing were not separated based on the isolation media used. Third-generation cephalosporin resistance was defined as ‘resistant to ampicillin, plus ceftazidime and/or cefotaxime in the disc diffusion test’ [50], but cephalosporin resistance was not defined. Since this resistance outcome represents a combination of antimicrobial classes classified under categories I and II of importance to human medicine [24], it has been placed after factors for which a potential association with fluoroquinolone (category I) [24] resistance was reported.

All references reported factors applied or present at the farm (Tables 1–4). One study also reported factors present at the hatchery [47]. Most references reported factors in commercial turkeys alone (*n* = 11; 85%). One reference reported data for breeder turkeys [49] and one reference reported data for both commercial and breeder turkeys [50].

### Synthesis of results: types of factors identified

In total, 36 unique factors were identified (Tables 1–4). Most factors (*n* = 27; 75%) were in commercial turkeys and the remainder (*n* = 9; 25%) were in breeder turkeys. Five themes emerged from the factors identified: (1) AMU policy change (Table 1); (2) binary qualitative AMU (i.e. AMU reported as a binary factor; yes or no), where information on dosing was not reported (e.g. tylosin use (preventive in-feed) *vs.* no tylosin use (preventive in-feed)) (Table 1); (3) binary quantitative AMU, where information on dosing was reported (e.g. continuous chlortetracycline use (200 mg/kg) administered in-feed *vs.* no AMU via feed or water) (Table 2); (4) biosecurity (Table 3) and (5) management practices (Table 4). Almost half of the factors belonged to the AMU themes (*n* = 15; 42%), followed by biosecurity (*n* = 11, 31%) and management practices (*n* = 10; 28%). Commercial turkey factors (*n* = 27) most frequently belonged to AMU themes (*n* = 13; 48%), followed by biosecurity (*n* = 7; 26%) and management practices (*n* = 7; 26%) (Tables 1–4). For breeder turkey factors (*n* = 9), biosecurity (*n* = 4; 44%) and management practices (*n* = 3; 33%) were the most frequent themes.

Within the binary qualitative and binary quantitative AMU factors in commercial turkeys (Tables 1 and 2), unspecified AMU was the most frequently reported factor (*n* = 3 references), followed by enrofloxacin use, tetracycline (tetracycline and chlortetracycline) use, tylosin use and virginiamycin use (*n* = 2 references each).

Within the binary qualitative AMU theme, the route of administration was either in-feed or unspecified (Table 1). Some binary qualitative AMU factors reported the reason for AMU (e.g. preventive, treatment), and/or whether the use occurred within a defined time period (e.g. within the last year). All binary quantitative AMU factors specified the dose, duration of use, temporal pattern of use (e.g. pulsed, continuous) and route of administration (Table 2). Within the binary quantitative AMU theme, drinking water was the most common route of administration, with the remainder administered in-feed.

Biosecurity factors (*n* = 11) included exposure to other animal populations (*n* = 5), specific cleaning and disinfection or hygiene practices (*n* = 4) and water supply (*n* = 2) (Table 3). Within the identified management practices, organic *vs.* conventional production was the most common factor, appearing in four references (Table 4). Three different management practice factors pertained to housing, while the remaining factors in this theme encompassed a variety of practices, including ‘raised without antibiotics’ production and number of birds on the farm.

### Resistance outcomes investigated

The resistance outcomes investigated in potential associations with identified factors are summarised in Tables 1–4. Out of the included references (*n* = 13), data were reported for AMR in *E. coli* (*n* = 9; 69%), *Campylobacter* species (*n* = 4; 31%, including references reporting *Campylobacter coli*, *Campylobacter jejuni* and *Campylobacter* species), and *Enterococcus* species (*n* = 3; 23%, including references reporting *Enterococcus faecalis*, *Enterococcus faecium* and *Enterococcus* species) (Tables 1–4). None of the identified references reported potential associations between factors and antimicrobial-resistant *S. enterica*.

Potential associations between factors and AMR were more commonly reported for the following resistance outcomes: aminoglycosides (*n* = 9; 69%), fluoroquinolones (*n* = 9; 69%), macrolides (*n* = 7; 54%), tetracyclines (*n* = 7; 54%), penicillins (*n* = 6; 46%) and other quinolones (*n* = 6; 46%) (Tables 1–4). Within each bacterial species, the most frequently reported resistance outcomes were as follows: *E. coli* (*n* = 9): aminoglycosides (*n* = 6; 67%) and fluoroquinolones (*n* = 6; 67%), *Campylobacter* species (*n* = 4): macrolides (*n* = 4; 100%) and *Enterococcus* species (*n* = 3): streptogramins (*n* = 3; 100%). Overall, the bacteria-resistance outcome combinations with the highest frequencies of reporting were *E. coli*/aminoglycoside (*n* = 6; 46%), *E. coli*/fluoroquinolone (*n* = 6; 46%), *E. coli*/penicillin (*n* = 5; 38%), *E. coli*/tetracycline (*n* = 5; 38%), *Campylobacter* species/macrolide (*n* = 4; 31%), *E. coli*/other quinolone (*n* = 4; 31%) and *E. coli*/cephalosporin (*n* = 4; 31%) (Tables 1–4).

## Discussion

This review identified factors potentially linked with antimicrobial-resistant *Campylobacter* species, *Enterococcus* species and *E. coli* from turkeys. Identified factors spanned AMU themes, as well as non-AMU themes (biosecurity and management practices). This review also revealed important data gaps; no factors pertaining to *S. enterica* or to stages in the farm-to-fork pathway other than the farm (e.g. abattoir, retail) were identified, and only one Canadian reference was found.

The final review included 13 references. There was limited depth in the literature, particularly within specific bacteria-resistance outcome combinations. This is consistent with findings from other reviews of factors potentially linked with AMR in livestock [16, 20]. The most common theme type was AMU. This was expected, as AMU is a well-known driver of AMR. Organic compared to conventional production was a predominant factor and there was considerable breadth in the identified non-AMU factors, findings observed in similar reviews [16, 20].

There were substantial data gaps regarding modifiable factors potentially linked with AMR in turkey production. For one, no factors were identified after the farm stage (e.g. abattoir and retail). Interventions after the farm stage may be responsible for the largest reductions in foodborne illness, including antimicrobial-resistant foodborne illness [52, 53]. Others have reported that interventions across multiple stages may be best for preventing foodborne illness from poultry consumption [53]. Our review did not include factors related to the burden of foodborne illness of turkey origin, as these were beyond the scope of this review. However, it is possible that current food safety pathogen reduction practices at the abattoir also influence AMR and consequently antimicrobial-resistant foodborne illness [53, 54]. The role of other sites along the farm-to-fork pathway in human exposure to foodborne bacteria from turkeys, including resistant bacteria, was a substantial data gap.

Another important data gap was the absence of factors potentially associated with resistance in *S. enterica*. Non-typhoidal salmonellosis is nationally notifiable in Canada [55]. It has been estimated that 34–42% of foodborne non-typhoidal salmonellosis illnesses are linked to poultry consumption [56]. Also, *Salmonella* Reading have been isolated from Canadian turkey farms, and FoodNet Canada has reported genetic links between human cases of salmonellosis and turkey manure [22, 57]. Given the public health significance of human non-typhoidal salmonellosis attributed to poultry and human outbreaks linked with MDR *Salmonella* [10, 11], the absence of references reporting factors potentially linked with antimicrobial-resistant *Salmonella* was a substantial gap.

AMU is widely considered the main contributor to AMR [4, 6] and there were AMU-related data gaps from this review. Findings from CIPARS show that bacitracins, penicillins, tetracyclines, trimethoprim/sulphonamides and virginiamycin were commonly reported as used in Canadian turkey flocks [22, 31]. No references included in this review reported potential associations between trimethoprim/sulphonamide or penicillin use and AMR. Few references reported the other commonly used antimicrobials. However, these findings may have been impacted by criteria of this review that were shaped by the needs of the larger iAM.AMR project at this stage of model development, such as the pragmatic exclusion of continuous factors. The use of continuous quantitative AMU indicators in surveillance reporting provides additional granularity for understanding the impact of AMU (compared to categorical binary measurements), and the reporting of such indicators is becoming more common [31, 58]. The research team plans to incorporate continuous quantitative AMU indicators, in particular, into future iterations of the iAM.AMR project's model structure.

Legislative changes related to AMU were identified in one reference: (1) incentive tools, followed by (2) a mandatory ban of the preventive use of critically important antimicrobials [23, 39]. These factors – or industry-led AMU stewardship initiatives – require further study to determine their applicability to the Canadian context. Health Canada has classified antimicrobials by their importance to human medicine: category I (very high importance), category II (high importance), category III (medium importance) and category IV (low importance) [24]. The Turkey Farmers of Canada set timelines for an Antimicrobial Use Reduction Strategy (AMU Reduction Strategy) calling for voluntary elimination of the preventive use of: (1) category I (May 2014), (2) category II (December 2018) and (3) category III antimicrobials (May 2020, evaluation ongoing) [32, 59, 60]. These antimicrobials are still available for use in disease treatment [32]. As a result of the Canadian poultry industry's AMU Reduction Strategy, an expected shift in AMU administration from preventive (in-feed) to treatment (typically in water) was observed; there has been a decreasing trend in the percentage of turkey flocks reporting any in-feed AMU since 2014 [22, 31]. In addition, the Food and Drug Regulations were amended in 2018 to increase veterinary oversight of medically important antimicrobials [61, 62]. Further study is needed to discern the impact of both industry-driven and regulatory directives on the occurrence of AMR.

Biosecurity and management practices were the most commonly reported themes for breeder turkeys and were also reported in commercial turkeys. This was expected; flock health is particularly important to the breeder industry given its careful genetic selection and role in supplying hatching eggs for turkey meat production [63]. Biosecurity and management practices are stricter in Canadian breeder turkey flocks compared to commercial flocks [63, 64].

Specific management practices and biosecurity measures of potential relevance to Canadian commercial turkey production were identified in this review, including rearing turkeys in reused litter material, movement to new housing partway through a production cycle, the age of the youngest birds in the flock and disinfection of floors and walls at depopulation. However, several data gaps relevant to Canadian turkey production remain. The Turkey Farmers of Canada On-Farm Food Safety Program has biosecurity and flock care requirements (e.g. dry cleaning (litter and manure removal) between consecutive flocks in brooding barns) [33]. These requirements may impact flock health and by extension the need for AMU, and are critical components to AMR mitigation [16, 31, 65].

Certain production practices may lead to the transmission of bacteria, including resistant bacteria, between flocks. In Canada, turkeys may be moved to a grow-out barn after brooding, where dry cleaning is only required yearly [33, 66]. Additionally, some producers rear birds in multi-age facilities, and move birds still being fattened onto litter from other barns or areas previously used by lighter market weight birds after these lighter birds are slaughtered [33, 66]. These practices present opportunities for the transfer of bacteria persisting in the litter, including resistant bacteria, between flocks [67].

Disinfection of barn floors and walls was also a factor identified. Canadian turkey producers may disinfect barn surfaces between consecutive flocks after standard dry cleaning, as disinfection can further reduce the presence of microorganisms important to public health and animal health, including resistant bacteria [33]. The efficacy of disinfection has been shown to be dependent on the barn flooring type, cleaning procedure (e.g. wet cleaning *vs.* dry cleaning *vs.* disinfection) and bacteria (e.g. *E. coli vs. Salmonella*) [68].

Given the high cost of production practices, including new bedding material, and the environmental challenges posed by the extra waste generated from frequent litter replenishment [67], more research on the integrated effects of housing, multi-age rearing practices, litter management (e.g. replacement, litter amendment and storage of used litter), specific cleaning and disinfection procedures and implementation of downtime (duration between flock cycles) on bacterial prevalence and AMR is needed.

While this review filled an important gap in the literature regarding turkeys and AMR, its approach had limitations. This review only included references reporting AMR in individual bacterial species. Reports of AMR from metagenomics or where only resistance genes were reported (without bacterial species) were excluded. Next-generation sequencing technologies may improve the understanding of food systems as complex bacterial ecosystems and are thus expected to assist foodborne AMR surveillance [69]. For example, metagenomics can improve the quantification of AMR in diverse samples (e.g. animal faeces) through relative abundance estimates. However, metagenomics results cannot be linked with particular bacteria of public health relevance, which are often focus areas for models such as the iAM.AMR project's [69].

Furthermore, relevant references may not have been captured on account of the decision to use frequency thresholds in the search strategy (Supplementary Tables S1–S10). These constrained the number of citations captured, to reduce the number of ineligible results and the time necessary to complete relevance screening. As scoping reviews are broad by nature [70], there is often a need to constrain a search for these reasons, and an inability to capture all relevant references is an expected limitation of scoping reviews in general [71]. Automated screening tools have been developed using machine learning, and these are expected to help mitigate this limitation in the future [18, 72]. These tools were not available to the research team at the time the searches were conducted. Finally, human resources constrained this scoping review to English-language publications. However, it is unlikely that these limitations have substantially impacted the interpretation of the findings [19].

Given the paucity of turkey-specific studies identified, more research is needed to recommend specific practices to the Canadian turkey industry. It is considered best practice to base policy decisions on the findings of multiple studies examining similar research questions, summarised using systematic review and meta-analysis [18, 19]. Due to the lack of study replication for individual factors identified, areas of focus for systematic reviews cannot be recommended.

## Conclusions

Knowledge syntheses on factors potentially linked with AMR in turkeys are limited, as cattle, chickens and pigs are often the focus of such work [16, 20, 21, 25–29]. Research specific to turkeys is a priority in Canada, given the consumption of turkey at holidays and as deli meat [8, 9]. Therefore, there is a need for a structured, comprehensive and transparent synthesis of factors potentially associated with AMR in turkeys. This review identified AMU, biosecurity and management practice factors potentially linked with antimicrobial-resistant *Campylobacter* species, *Enterococcus* species and *E. coli* of turkey origin. No factors potentially associated with AMR in *Salmonella* were identified, and this should be a priority for future work. In addition, only one Canadian reference was found, and no factors pertaining to stages of the farm-to-fork pathway other than the farm were identified.

This scoping review is an important addition to the body of literature on foodborne AMR. The findings from this review will be incorporated into the larger iAM.AMR project to evaluate potential interventions for reducing the overall occurrence of AMR along the farm-to-fork pathway. Research on the impacts of Canadian industry practices on AMR is necessary to develop a better understanding of AMR in turkeys. Consumption of turkey meat is an important potential source of human exposure to foodborne bacteria, including resistant foodborne bacteria in Canada. The findings from this review (factors reported and data gaps) will play a key role in informing priorities for future research, surveillance and understanding of the epidemiology of AMR in turkey production and associated public health risks.

## Data Availability

The full set of data generated from this scoping review is available on request from the corresponding author.

## Supplementary material

For supplementary material accompanying this paper visit http://doi.org/10.1017/S0950268822001224. click here to view supplementary material
