# Supplementary material for: A scoping review of factors potentially linked with antimicrobial-resistant bacteria from turkeys (iAM.AMR Project)
Source: Epidemiol Infect. 2022 Jul 18;150:e153. doi: 10.1017/S0950268822001224 (PMC9428905; doi:10.1017/S0950268822001224)
Supplement: Supplementary file 1 [file S0950268822001224sup001.docx]

*Epidemiology and Infection*

A scoping review of factors potentially linked with antimicrobial-resistant bacteria from turkeys (iAM.AMR Project)

Charly Phillips, Brennan Chapman, Agnes Agunos, Carolee A. Carson, E. Jane Parmley, Richard J. Reid-Smith, Ben A. Smith, Colleen P. Murphy

**Supplementary Material**

**Supplementary Table S1.** Medline search for factors potentially associated with antimicrobial resistance in *Campylobacter* species, *Enterococcus* species, *Escherichia coli*, and *Salmonella enterica* from cattle, chicken, pigs, and turkeys^a^

| **#** | **Searches** | **Results** |
| --- | --- | --- |
| 1 | exp *Drug Resistance, Microbial/ or exp *Drug Resistance, Multiple/ | 70939 |
| 2 | ((resistan* adj4 (antimicrobial* or microbial* or antibiotic* or anti biotic* or antibacterial* or bacteria* or multidrug* or multi drug* or extensively drug or multiple drug* or multiclass* or multi class* or multiple class*)) or amr).ti,kf,kw. or ((resistan* adj4 (antimicrobial* or microbial* or antibiotic* or anti biotic* or antibacterial* or bacteria* or multidrug* or multi drug* or extensively drug or multiple drug* or multiclass* or multi class* or multiple class*)) or amr).ab. /freq=2 | 80816 |
| 3^b^ | exp *beta-Lactams/ or *tetracycline/ or exp *quinolones/ or exp *macrolides/ or exp *nalidixic acid/ or exp *ciprofloxacin/ or *enrofloxacin/ | 170367 |
| 4^b^ | ("B-lactam*" or "beta-lactam*" or penicillin* or carbapenem* or cephalosporin* or moxalactam* or latamoxef* or tetracycline or quinolone* or "4-Quinolone*" or fluoroquinolone* or macrolide* or tylosin* or ciprofloxacin* or enrofloxacin*).ti,ab,kw,kf. | 176942 |
| 5 | or/1-4 [AMR] | 354638 |
| 6 | *cattle/ or exp *swine/ or *chickens/ or *turkeys/ or (cattle or cow? or bull or bulls or steer? or calf or calves or bos taurus or beef or veal or pig? or piglet? or swine? or hog? or sow? or pork or sus scrofa domesticus or chick? or chicken? or rooster? or hen? or broiler? or gallus gallus domesticus or turkeys or meleagris gallopavo or turkey or gobbler? or poultr*).ti,kf,kw. or (cattle or cow? or bull or bulls or steer? or calf or calves or bos taurus or beef or veal or pig? or piglet? or swine? or hog? or sow? or pork or sus scrofa domesticus or chick? or chicken? or rooster? or hen? or broiler? or gallus gallus domesticus or turkeys or meleagris gallopavo or turkey or gobbler? or poultr*).ab. /freq=2 | 494578 |
| 7 | exp *Escherichia coli/ or (Escherichia coli or "e coli" or ecoli).ti,kf,kw. or (Escherichia coli or "e coli" or ecoli).ab. /freq=2 | 199672 |
| 8 | exp *salmonella/ or salmonella.ti,kw,kf. or salmonella.ab. /freq=2 | 56026 |
| 9 | exp *Campylobacter/ or campylobacter.ti,kw,kf. or campylobacter.ab. /freq=2 | 12470 |
| 10 | *Enterococcus faecium/ or (enterococcus faecium or "e faecium").ti,kw,kf. or (enterococcus faecium or "e faecium").ab. /freq=2 | 3887 |
| 11 | *Enterococcus faecalis/ or (Enterococcus faecalis or "e faecalis").ti,kw,kf. or (Enterococcus faecalis or "e faecalis").ab. /freq=2 | 8213 |
| 12 | or/7-11 | 270276 |
| 13 | and/5-6,12 | 3834 |

^a^The dates of coverage for this database were 1946 to April 10, 2019.

^b^Search terms for extended-spectrum cephalosporins, quinolones, macrolides, and tetracyclines were added to improve the sensitivity of the search for these antimicrobial classes. These classes are of particular relevance to the iAM.AMR project [1].

**Supplementary Table S2.** Medline search for factors potentially associated with antimicrobial resistance in *Campylobacter* species, *Enterococcus* species, *Escherichia coli*, and *Salmonella enterica* from turkeys^a,b^

| **#** | **Searches** | **Results** |
| --- | --- | --- |
| 1 | exp *Drug Resistance, Microbial/ or exp *Drug Resistance, Multiple/ | 80471 |
| 2 | ((resistan* adj4 (antimicrobial* or microbial* or antibiotic* or anti biotic* or antibacterial* or bacteria* or multidrug* or multi drug* or extensively drug or multiple drug* or multiclass* or multi class* or multiple class*)) or amr).ti,kf,kw. or ((resistan* adj4 (antimicrobial* or microbial* or antibiotic* or anti biotic* or antibacterial* or bacteria* or multidrug* or multi drug* or extensively drug or multiple drug* or multiclass* or multi class* or multiple class*)) or amr).ab. /freq=2 | 105009 |
| 3^c^ | exp *beta-Lactams/ or *tetracycline/ or exp *quinolones/ or exp *macrolides/ or exp *nalidixic acid/ or exp *ciprofloxacin/ or *enrofloxacin/ | 182359 |
| 4^c^ | ("B-lactam*" or "beta-lactam*" or penicillin* or carbapenem* or cephalosporin* or moxalactam* or latamoxef* or tetracycline or quinolone* or "4-Quinolone*" or fluoroquinolone* or macrolide* or tylosin* or ciprofloxacin* or enrofloxacin*).ti,ab,kw,kf. | 200229 |
| 5 | or/1-4 [AMR] | 403015 |
| 6 | *turkeys/ or (turkeys or meleagris gallopavo or turkey or gobbler?).ti,kf,kw. or (turkeys or meleagris gallopavo or turkey or gobbler?).ab. /freq=2 | 26061 |
| 7 | exp *Escherichia coli/ or (Escherichia coli or "e coli" or ecoli).ti,kf,kw. or (Escherichia coli or "e coli" or ecoli).ab. /freq=2 | 216903 |
| 8 | exp *salmonella/ or salmonella.ti,kw,kf. or salmonella.ab. /freq=2 | 61003 |
| 9 | exp *Campylobacter/ or campylobacter.ti,kw,kf. or campylobacter.ab. /freq=2 | 13699 |
| 10 | *Enterococcus faecium/ or (enterococcus faecium or "e faecium").ti,kw,kf. or (enterococcus faecium or "e faecium").ab. /freq=2 | 4544 |
| 11 | *Enterococcus faecalis/ or (Enterococcus faecalis or "e faecalis").ti,kw,kf. or (Enterococcus faecalis or "e faecalis").ab. /freq=2 | 9441 |
| 12 | or/7-11 | 294281 |
| 13 | and/5-6,12 | 334 |
| 14 | (201904* or 201905* or 201906* or 201907* or 201908* or 201909* or 20191* or 202*).ez,dt,ed. | 4174847 |
| 15 | 13 and 14 | 62 |
| 16 | Turkey/ | 37342 |
| 17 | (adana or adiyaman or afyonkarahisar or agri or amasya or ankara or antalya or artvin or aydin or balikesir or bilecik or bingol or bitlis or bolu or burdur or bursa or canakkale or cankiri or corum or denizli or diyarbakir or edirne or elazig or erzincan or erzurum or eskisehir or gaziantep or giresun or gumushane or hakkari or hatay or isparta or mersin or istanbul or izmir or kars or kastamonu or kayseri or kirklareli or kirsehir or kocaeli or konya or kutahya or malatya or manisa or kahramanmaras or mardin or mugla or nevsehir or nigde or ordu or rize or sakarya or samsun or siirt or sinop or sivas or tekirdag or tokat or trabzon or tunceli or sanliurfa or usak or yozgat or zonguldak or aksaray or bayburt or karaman or kirikkale or batman or sirnak or bartin or ardahan or igdir or yalova or karabuk or kilis or osmaniye or duzce).tw,kf,kw. | 25043 |
| 18 | 16 or 17 | 54195 |
| 19 | 15 not 18 | 46 |

^a^The initial search was also altered to filter out terms related to Turkey (the country).

^b^The dates of coverage for this database were 1946 to August 10, 2021.

^c^Search terms for extended-spectrum cephalosporins, quinolones, macrolides, and tetracyclines were added to improve the sensitivity of the search for these antimicrobial classes. These classes are of particular relevance to the iAM.AMR project [1].

**Supplementary Table S3**. Embase search for factors potentially associated with antimicrobial resistance in *Campylobacter* species, *Enterococcus* species, *Escherichia coli*, and *Salmonella enterica* from cattle, chicken, pigs, and turkeys^a^

| **#** | **Searches** | **Results** |
| --- | --- | --- |
| 1 | exp *antibiotic resistance/ or *multidrug resistance/ or *cross resistance/ | 68099 |
| 2 | ((resistan* adj4 (antimicrobial* or microbial* or antibiotic* or anti biotic* or antibacterial* or bacteria* or multidrug* or multi drug* or extensively drug or multiple drug* or multiclass* or multi class* or multiple class*)) or amr).ti,kw. or ((resistan* adj4 (antimicrobial* or microbial* or antibiotic* or anti biotic* or antibacterial* or bacteria* or multidrug* or multi drug* or extensively drug or multiple drug* or multiclass* or multi class* or multiple class*)) or amr).ab. /freq=2 | 102496 |
| 3^b^ | *beta lactam/ or *beta lactam antibiotic/ or *beta-lactam resistance/ or exp *penicillin derivative/ or exp *penicillin resistance/ or *carbapenem/ or *carbapenem derivative/ or *cephalosporin derivative/ or *cephalosporin resistance/ or *quinolone derivative/ or exp *macrolide/ or *ciprofloxacin/ | 175250 |
| 4^b^ | ("B-lactam*" or "beta-lactam*" or penicillin* or carbapenem* or cephalosporin* or moxalactam* or latamoxef* or tetracycline* or quinolone* or "4-Quinolone*" or fluoroquinolone* or macrolide* or tylosin* or ciprofloxacin* or enrofloxacin*).ti,ab,kw. | 203229 |
| 5 | or/1-4 | 397288 |
| 6 | (exp *bovine/ or exp *pig/ or exp *chicken/ or exp *"turkey (bird)"/) and *meat/ | 948 |
| 7 | *chicken meat/ or *turkey meat/ or *pork/ or *veal/ or *beef/ or (broiler? or beef or veal or pork or bull or bulls or steer? or pig? or piglet? or swine? or hog? or sow? or turkeys or meleagris gallopavo or turkey or gobbler? or rooster? or sus scrofa domesticus or bos taurus or gallus gallus domesticus or cattle or cow? or calf or calves or chick? or chicken? or hen? or poultr*).ti,kw. or (broiler? or beef or veal or pork or bull or bulls or steer? or pig? or piglet? or swine? or hog? or sow? or turkeys or meleagris gallopavo or turkey or gobbler? or rooster? or sus scrofa domesticus or bos taurus or gallus gallus domesticus or cattle or cow? or calf or calves or chick? or chicken? or hen? or poultr*).ab. /freq=2 | 457406 |
| 8 | 6 or 7 | 457453 |
| 9 | exp *Escherichia coli/ or (Escherichia coli or "e coli" or ecoli).ti,kw. or (Escherichia coli or "e coli" or ecoli).ab. /freq=2 | 200664 |
| 10 | exp *salmonella/ or salmonella.ti,kw. or salmonella.ab. /freq=2 | 49288 |
| 11 | exp *Campylobacter/ or campylobacter.ti,kw. or campylobacter.ab. /freq=2 | 13929 |
| 12 | *Enterococcus faecium/ or (enterococcus faecium or "e faecium").ti,kw. or (enterococcus faecium or "e faecium").ab. /freq=2 | 4305 |
| 13 | *Enterococcus faecalis/ or (Enterococcus faecalis or "e faecalis").ti,kw. or (Enterococcus faecalis or "e faecalis").ab. /freq=2 | 9122 |
| 14 | or/9-13 | 266000 |
| 15 | and/5,8,14 | 3985 |

^a^The dates of coverage for this database were 1974 to April 9, 2019.

^b^Search terms for extended-spectrum cephalosporins, quinolones, macrolides, and tetracyclines were added to improve the sensitivity of the search for these antimicrobial classes. These classes are of particular relevance to the iAM.AMR project [1].

**Supplementary Table S4.** Agricola search for factors potentially associated with antimicrobial resistance in *Campylobacter* species, *Enterococcus* species, *Escherichia coli*, and *Salmonella enterica* from cattle, chicken, pigs, and turkeys^a^

| **#** | **Searches** | **Results** |
| --- | --- | --- |
| 1 | antibiotic resistance/ or multiple drug resistance/ | 7019 |
| 2 | ((resistan* adj4 (antimicrobial* or microbial* or antibiotic* or anti biotic* or antibacterial* or bacteria* or multidrug* or multi drug* or extensively drug or multiple drug* or multiclass* or multi class* or multiple class*)) or amr).tw,hw. | 23658 |
| 3^b^ | beta-lactam antibiotics/ or beta-lactamase/ or exp penicillins/ or exp cephalosporins/ or exp quinolones/ or exp tetracyclines/ or exp macrolides/ | 10362 |
| 4^b^ | ("b-lactam*" or "beta-lactam*" or penicillin* or carbapenem* or cephalosporin* or moxalactam* or latamoxef* or tetracycline* or quinolone* or "4-Quinolone*" or fluoroquinolone* or macrolide* or tylosin* or ciprofloxacin* or enrofloxacin*).tw,hw. | 17762 |
| 5 | or/1-4 | 39252 |
| 6 | (exp cattle/ or exp swine/ or exp chickens/ or exp turkeys/) and (meat/ or exp meat products/ or meat production/) | 7213 |
| 7 | exp chicken meat/ or exp poultry skin/ or exp turkey meat/ or exp pork/ or exp beef/ or (cattle or cow? or bull or bulls or steer? or calf or calves or bos taurus or beef or veal or pig? or piglet? or swine? or hog? or sow? or pork or sus scrofa domesticus or chick? or chicken? or rooster? or hen? or broiler? or gallus gallus domesticus or turkeys or meleagris gallopavo or turkey or gobbler? or poultr*).tw,hw. | 507940 |
| 8 | 6 or 7 | 507955 |
| 9 | exp escherichia coli/ or (escherichia coli or "e coli" or ecoli).tw,hw. | 64875 |
| 10 | exp salmonella/ or salmonella.tw,hw. | 22156 |
| 11 | exp campylobacter/ or campylobacter.tw,hw. | 4894 |
| 12 | enterococcus faecium/ or (enterococcus faecium or "e faecium").tw,hw. | 1816 |
| 13 | enterococcus faecalis/ or (enterococcus faecalis or "e faecalis").tw,hw. | 2410 |
| 14 | or/9-13 | 87187 |
| 15 | and/5,8,14 | 2525 |

^a^The dates of coverage for this database were 1970 to March 2019.

^b^Search terms for extended-spectrum cephalosporins, quinolones, macrolides, and tetracyclines were added to improve the sensitivity of the search for these antimicrobial classes. These classes are of particular relevance to the iAM.AMR project [1].

**Supplementary Table S5**. CAB Abstracts search for factors potentially associated with antimicrobial resistance in *Campylobacter* species, *Enterococcus* species, *Escherichia coli*, and *Salmonella enterica* from cattle, chicken, pigs, and turkeys^a^

| **#** | **Searches** | **Results** |
| --- | --- | --- |
| 1 | exp drug resistance/ | 55766 |
| 2 | ((resistan* adj4 (antimicrobial* or microbial* or antibiotic* or anti biotic* or antibacterial* or bacteria* or multidrug* or multi drug* or extensively drug or multiple drug* or multiclass* or multi class* or multiple class*)) or amr).ti,hw. or ((resistan* adj4 (antimicrobial* or microbial* or antibiotic* or anti biotic* or antibacterial* or bacteria* or multidrug* or multi drug* or extensively drug or multiple drug* or multiclass* or multi class* or multiple class*)) or amr).ab. /freq=2 | 29968 |
| 3^b^ | exp beta-lactam antibiotics/ or latamoxef/ or exp tetracyclines/ or quinolones/ or macrolide antibiotics/ or exp fluoroquinolone antibiotics/ | 65859 |
| 4^b^ | ("b-lactam*" or "beta-lactam*" or penicillin* or carbapenem* or cephalosporin* or moxalactam* or latamoxef* or tetracycline* or quinolone* or "4-Quinolone*" or fluoroquinolone* or macrolide* or tylosin* or ciprofloxacin* or enrofloxacin*).ti,hw. or ("b-lactam*" or "beta-lactam*" or penicillin* or carbapenem* or cephalosporin* or moxalactam* or latamoxef* or tetracycline* or quinolone* or "4-Quinolone*" or fluoroquinolone* or macrolide* or tylosin* or ciprofloxacin* or enrofloxacin*).ab. /freq=2 | 68372 |
| 5 | or/1-4 | 110329 |
| 6 | (meat/ or exp meat cuts/ or exp meat products/) and (exp cattle/ or exp pigs/ or exp turkeys/ or exp fowls/) | 18945 |
| 7 | exp beef cattle/ or exp pigmeat/ or chicken meat/ or turkey meat/ or (broiler? or beef or pork or veal or bos taurus or sus scrofa domesticus or gallus gallus domesticus or meleagris gallopavo).ti,hw. or (broiler? or beef or pork or veal or bos taurus or sus scrofa domesticus or gallus gallus domesticus or meleagris gallopavo).ab. /freq=2 | 146697 |
| 8 | ((cattle or cow? or bull or bulls or steer? or calf or calves or pig? or piglet? or swine? or hog? or sow? or chick? or chicken? or rooster? or hen? or turkeys or turkey or gobbler? or poultr*) adj5 (meat? or food or foods or consum* or eat or eaten or slaughter* or butcher*)).ti,hw. or ((cattle or cow? or bull or bulls or steer? or calf or calves or pig? or piglet? or swine? or hog? or sow? or chick? or chicken? or rooster? or hen? or turkeys or turkey or gobbler? or poultr*) adj5 (meat? or food or foods or consum* or eat or eaten or slaughter* or butcher*)).ab. /freq=2 | 50286 |
| 9 | or/6-8 | 177656 |
| 10 | exp escherichia coli/ or (escherichia coli or "e coli" or ecoli).ti,hw. or (escherichia coli or "e coli" or ecoli).ab. /freq=2 | 95967 |
| 11 | exp salmonella/ or salmonella.ti,hw. or salmonella.ab. /freq=2 | 52993 |
| 12 | exp campylobacter/ or campylobacter.ti,hw. or campylobacter.ab. /freq=2 | 11468 |
| 13 | enterococcus faecium/ or (enterococcus faecium or "e faecium").ti,hw. or (enterococcus faecium or "e faecium").ab. /freq=2 | 4085 |
| 14 | enterococcus faecalis/ or (enterococcus faecalis or "e faecalis").ti,hw. or (enterococcus faecalis or "e faecalis").ab. /freq=2 | 6655 |
| 15 | or/10-14 | 145742 |
| 16 | and/5,9,15 | 2893 |

^a^The dates of coverage for this database were 1973 to 2019 Week 13.

^b^Search terms for extended-spectrum cephalosporins, quinolones, macrolides, and tetracyclines were added to improve the sensitivity of the search for these antimicrobial classes. These classes are of particular relevance to the iAM.AMR project [1].

**Supplementary Table S6.** Food Science and Technology Abstracts search for factors potentially associated with antimicrobial resistance in *Campylobacter* species, *Enterococcus* species, *Escherichia coli*, and *Salmonella enterica* from cattle, chicken, pigs, and turkeys^a^

| **#** | **Searches** | **Results** |
| --- | --- | --- |
| 1 | ANTIBIOTICS RESISTANCE/ | 6095 |
| 2 | ((resistan* adj4 (antimicrobial* or microbial* or antibiotic* or anti biotic* or antibacterial* or bacteria* or multidrug* or multi drug* or extensively drug or multiple drug* or multiclass* or multi class* or multiple class*)) or amr).tw,hw. | 10433 |
| 3^b^ | beta-LACTAM ANTIBIOTICs/ or beta-LACTAMASES/ or exp TETRACYCLINES/ or exp QUINOLONES/ or exp MACROLIDE ANTIBIOTICS/ | 2519 |
| 4^b^ | ("b-lactam*" or "beta-lactam*" or penicillin* or carbapenem* or cephalosporin* or moxalactam* or latamoxef* or tetracycline* or quinolone* or "4-Quinolone*" or fluoroquinolone* or macrolide* or tylosin* or ciprofloxacin* or enrofloxacin*).tw,hw. | 8095 |
| 5 | or/1-4 | 14975 |
| 6 | (exp meat/ or meat products/) and (exp cattle/ or exp swine/ or chickens/ or turkeys/) | 20903 |
| 7 | exp beef/ or exp veal/ or exp pork/ or exp chicken meat/ or exp turkey meat/ or (broiler? or beef or pork or veal or bos taurus or sus scrofa domesticus or gallus gallus domesticus or meleagris gallopavo).tw,hw. | 73072 |
| 8 | (cattle or cow? or bull or bulls or steer? or calf or calves or pig? or piglet? or swine? or hog? or sow? or chick? or chicken? or rooster? or hen? or turkeys or turkey or gobbler? or poultr*).ti,hw. or (cattle or cow? or bull or bulls or steer? or calf or calves or pig? or piglet? or swine? or hog? or sow? or chick? or chicken? or rooster? or hen? or turkeys or turkey or gobbler? or poultr*).ab. /freq=2 | 90981 |
| 9 | or/6-8 | 121712 |
| 10 | ESCHERICHIA COLI/ or (escherichia coli or "e coli" or ecoli).tw,hw. | 36688 |
| 11 | exp SALMONELLA/ or salmonella.tw,hw. | 25417 |
| 12 | exp campylobacter/ or campylobacter.tw,hw. | 5666 |
| 13 | ENTEROCOCCUS FAECIUM/ or (enterococcus faecium or "e faecium").tw,hw. | 1637 |
| 14 | ENTEROCOCCUS FAECALIS/ or (enterococcus faecalis or "e faecalis").tw,hw. | 1740 |
| 15 | or/10-14 | 59654 |
| 16 | and/5,9,15 | 2366 |

^a^The dates of coverage for this database were 1969 to 2019 April Week 1.

^b^Search terms for extended-spectrum cephalosporins, quinolones, macrolides, and tetracyclines were added to improve the sensitivity of the search for these antimicrobial classes. These classes are of particular relevance to the iAM.AMR project [1].

**Supplementary Table S7.** Embase search for factors potentially associated with antimicrobial resistance in *Campylobacter* species, *Enterococcus* species, *Escherichia coli*, and *Salmonella enterica* from turkeys^a,b^

| **#** | **Searches** | **Results** |
| --- | --- | --- |
| 1 | exp *antibiotic resistance/ or *multidrug resistance/ or *cross resistance/ | 81576 |
| 2 | ((resistan* adj4 (antimicrobial* or microbial* or antibiotic* or anti biotic* or antibacterial* or bacteria* or multidrug* or multi drug* or extensively drug or multiple drug* or multiclass* or multi class* or multiple class*)) or amr).ti,kw. or ((resistan* adj4 (antimicrobial* or microbial* or antibiotic* or anti biotic* or antibacterial* or bacteria* or multidrug* or multi drug* or extensively drug or multiple drug* or multiclass* or multi class* or multiple class*)) or amr).ab. /freq=2 | 131738 |
| 3^c^ | *beta lactam/ or *beta lactam antibiotic/ or *beta-lactam resistance/ or exp *penicillin derivative/ or exp *penicillin resistance/ or *carbapenem/ or *carbapenem derivative/ or *cephalosporin derivative/ or *cephalosporin resistance/ or *quinolone derivative/ or exp *macrolide/ or *ciprofloxacin/ | 204267 |
| 4^c^ | ("B-lactam*" or "beta-lactam*" or penicillin* or carbapenem* or cephalosporin* or moxalactam* or latamoxef* or tetracycline* or quinolone* or "4-Quinolone*" or fluoroquinolone* or macrolide* or tylosin* or ciprofloxacin* or enrofloxacin*).ti,ab,kw. | 234806 |
| 5 | or/1-4 | 471901 |
| 6 | *"turkey (bird)"/ and *meat/ | 92 |
| 7 | *turkey meat/ or (turkeys or meleagris gallopavo or turkey or gobbler?).ti,kw. or (turkeys or meleagris gallopavo or turkey or gobbler?).ab. /freq=2 | 31491 |
| 8 | 6 or 7 | 31494 |
| 9 | exp *Escherichia coli/ or (Escherichia coli or "e coli" or ecoli).ti,kw. or (Escherichia coli or "e coli" or ecoli).ab. /freq=2 | 218142 |
| 10 | exp *salmonella/ or salmonella.ti,kw. or salmonella.ab. /freq=2 | 54271 |
| 11 | exp *Campylobacter/ or campylobacter.ti,kw. or campylobacter.ab. /freq=2 | 15152 |
| 12 | *Enterococcus faecium/ or (enterococcus faecium or "e faecium").ti,kw. or (enterococcus faecium or "e faecium").ab. /freq=2 | 5069 |
| 13 | *Enterococcus faecalis/ or (Enterococcus faecalis or "e faecalis").ti,kw. or (Enterococcus faecalis or "e faecalis").ab. /freq=2 | 10580 |
| 14 | or/9-13 | 290544 |
| 15 | and/5,8,14 | 361 |
| 16 | (201904* or 201905* or 201906* or 201907* or 201908* or 201909* or 20191* or 202*).dc,dd. | 4785910 |
| 17 | 15 and 16 | 62 |
| 18 | (adana or adiyaman or afyonkarahisar or agri or amasya or ankara or antalya or artvin or aydin or balikesir or bilecik or bingol or bitlis or bolu or burdur or bursa or canakkale or cankiri or corum or denizli or diyarbakir or edirne or elazig or erzincan or erzurum or eskisehir or gaziantep or giresun or gumushane or hakkari or hatay or isparta or mersin or istanbul or izmir or kars or kastamonu or kayseri or kirklareli or kirsehir or kocaeli or konya or kutahya or malatya or manisa or kahramanmaras or mardin or mugla or nevsehir or nigde or ordu or rize or sakarya or samsun or siirt or sinop or sivas or tekirdag or tokat or trabzon or tunceli or sanliurfa or usak or yozgat or zonguldak or aksaray or bayburt or karaman or kirikkale or batman or sirnak or bartin or ardahan or igdir or yalova or karabuk or kilis or osmaniye or duzce).tw,kw. | 36132 |
| 19 | (((north* or east* or south* or west*) adj turkey) or turkish).tw,kw. | 31495 |
| 20 | 18 or 19 | 63857 |
| 21 | 17 not 20 | 47 |

^a^The initial search was also altered to filter out terms related to Turkey (the country).

^b^The dates of coverage for this database were 1974 to August 10, 2021.

^c^Search terms for extended-spectrum cephalosporins, quinolones, macrolides, and tetracyclines were added to improve the sensitivity of the search for these antimicrobial classes. These classes are of particular relevance to the iAM.AMR project [1].

**Supplementary Table S8**. Agricola search for factors potentially associated with antimicrobial resistance in *Campylobacter* species, *Enterococcus* species, *Escherichia coli*, and *Salmonella enterica* from turkeys^a,b^

| **#** | **Searches** | **Results** |
| --- | --- | --- |
| 1 | antibiotic resistance/ or multiple drug resistance/ | 21182 |
| 2 | ((resistan* adj4 (antimicrobial* or microbial* or antibiotic* or anti biotic* or antibacterial* or bacteria* or multidrug* or multi drug* or extensively drug or multiple drug* or multiclass* or multi class* or multiple class*)) or amr).tw,hw. | 33063 |
| 3^c^ | beta-lactam antibiotics/ or beta-lactamase/ or exp penicillins/ or exp cephalosporins/ or exp quinolones/ or exp tetracyclines/ or exp macrolides/ | 30441 |
| 4^c^ | ("b-lactam*" or "beta-lactam*" or penicillin* or carbapenem* or cephalosporin* or moxalactam* or latamoxef* or tetracycline* or quinolone* or "4-Quinolone*" or fluoroquinolone* or macrolide* or tylosin* or ciprofloxacin* or enrofloxacin*).tw,hw. | 24120 |
| 5 | or/1-4 | 58874 |
| 6 | exp turkeys/ and (meat/ or exp meat products/ or meat production/) | 396 |
| 7 | exp turkey meat/ or (turkeys or meleagris gallopavo or turkey or gobbler?).tw,hw. | 27477 |
| 8 | 6 or 7 | 27477 |
| 9 | exp escherichia coli/ or (escherichia coli or "e coli" or ecoli).tw,hw. | 75763 |
| 10 | exp salmonella/ or salmonella.tw,hw. | 25451 |
| 11 | exp campylobacter/ or campylobacter.tw,hw. | 5413 |
| 12 | enterococcus faecium/ or (enterococcus faecium or "e faecium").tw,hw. | 2183 |
| 13 | enterococcus faecalis/ or (enterococcus faecalis or "e faecalis").tw,hw. | 3017 |
| 14 | or/9-13 | 100886 |
| 15 | and/5,8,14 | 262 |
| 16 | (201904* or 201905* or 201906* or 201907* or 201908* or 201909* or 20191* or 202*).up. | 7815946 |
| 17 | 15 and 16 | 262 |
| 18 | turkey.in. | 29878 |
| 19 | (adana or adiyaman or afyonkarahisar or agri or amasya or ankara or antalya or artvin or aydin or balikesir or bilecik or bingol or bitlis or bolu or burdur or bursa or canakkale or cankiri or corum or denizli or diyarbakir or edirne or elazig or erzincan or erzurum or eskisehir or gaziantep or giresun or gumushane or hakkari or hatay or isparta or mersin or istanbul or izmir or kars or kastamonu or kayseri or kirklareli or kirsehir or kocaeli or konya or kutahya or malatya or manisa or kahramanmaras or mardin or mugla or nevsehir or nigde or ordu or rize or sakarya or samsun or siirt or sinop or sivas or tekirdag or tokat or trabzon or tunceli or sanliurfa or usak or yozgat or zonguldak or aksaray or bayburt or karaman or kirikkale or batman or sirnak or bartin or ardahan or igdir or yalova or karabuk or kilis or osmaniye or duzce).tw,hw. | 12910 |
| 20 | (((north* or east* or south* or west*) adj turkey) or turkish).tw,hw. | 4850 |
| 21 | 18 or 19 or 20 | 41799 |
| 22 | 17 not 21 | 207 |

^a^The initial search was also altered to filter out terms related to Turkey (the country).

^b^The dates of coverage for this database were 1970 to July 2021.

^c^Search terms for extended-spectrum cephalosporins, quinolones, macrolides, and tetracyclines were added to improve the sensitivity of the search for these antimicrobial classes. These classes are of particular relevance to the iAM.AMR project [1].

**Supplementary Table S9.** CAB Abstracts search for factors potentially associated with antimicrobial resistance in *Campylobacter* species, *Enterococcus* species, *Escherichia coli*, and *Salmonella enterica* from turkeys^a,b^

| **#** | **Searches** | **Results** |
| --- | --- | --- |
| 1 | exp drug resistance/ | 69801 |
| 2 | ((resistan* adj4 (antimicrobial* or microbial* or antibiotic* or anti biotic* or antibacterial* or bacteria* or multidrug* or multi drug* or extensively drug or multiple drug* or multiclass* or multi class* or multiple class*)) or amr).ti,hw. or ((resistan* adj4 (antimicrobial* or microbial* or antibiotic* or anti biotic* or antibacterial* or bacteria* or multidrug* or multi drug* or extensively drug or multiple drug* or multiclass* or multi class* or multiple class*)) or amr).ab. /freq=2 | 39868 |
| 3^c^ | exp beta-lactam antibiotics/ or latamoxef/ or exp tetracyclines/ or quinolones/ or macrolide antibiotics/ or exp fluoroquinolone antibiotics/ | 78472 |
| 4^c^ | ("b-lactam*" or "beta-lactam*" or penicillin* or carbapenem* or cephalosporin* or moxalactam* or latamoxef* or tetracycline* or quinolone* or "4-Quinolone*" or fluoroquinolone* or macrolide* or tylosin* or ciprofloxacin* or enrofloxacin*).ti,hw. or ("b-lactam*" or "beta-lactam*" or penicillin* or carbapenem* or cephalosporin* or moxalactam* or latamoxef* or tetracycline* or quinolone* or "4-Quinolone*" or fluoroquinolone* or macrolide* or tylosin* or ciprofloxacin* or enrofloxacin*).ab. /freq=2 | 82055 |
| 5 | or/1-4 | 133946 |
| 6 | (meat/ or exp meat cuts/ or exp meat products/) and exp turkeys/ | 450 |
| 7 | turkey meat/ or meleagris gallopavo.ti,hw. or meleagris gallopavo.ab. /freq=2 | 2229 |
| 8 | ((turkeys or turkey or gobbler?) adj5 (meat? or food or foods or consum* or eat or eaten or slaughter* or butcher*)).ti,hw. or ((turkeys or turkey or gobbler?) adj5 (meat? or food or foods or consum* or eat or eaten or slaughter* or butcher*)).ab. /freq=2 | 3173 |
| 9 | or/6-8 | 3889 |
| 10 | exp escherichia coli/ or (escherichia coli or "e coli" or ecoli).ti,hw. or (escherichia coli or "e coli" or ecoli).ab. /freq=2 | 111670 |
| 11 | exp salmonella/ or salmonella.ti,hw. or salmonella.ab. /freq=2 | 59822 |
| 12 | exp campylobacter/ or campylobacter.ti,hw. or campylobacter.ab. /freq=2 | 12656 |
| 13 | enterococcus faecium/ or (enterococcus faecium or "e faecium").ti,hw. or (enterococcus faecium or "e faecium").ab. /freq=2 | 4924 |
| 14 | enterococcus faecalis/ or (enterococcus faecalis or "e faecalis").ti,hw. or (enterococcus faecalis or "e faecalis").ab. /freq=2 | 7987 |
| 15 | or/10-14 | 167375 |
| 16 | and/5,9,15 | 163 |
| 17 | (201904* or 201905* or 201906* or 201907* or 201908* or 201909* or 20191* or 202*).up. | 817061 |
| 18 | 16 and 17 | 20 |
| 19 | turkey.gl. | 77795 |
| 20 | 18 not 19 | 11 |

^a^The initial search was also altered to filter out terms related to Turkey (the country).

^b^The dates of coverage for this database were 1973 to 2021 Week 31.

^c^Search terms for extended-spectrum cephalosporins, quinolones, macrolides, and tetracyclines were added to improve the sensitivity of the search for these antimicrobial classes. These classes are of particular relevance to the iAM.AMR project [1].

**Supplementary Table S10.** Food Science and Technology Abstracts search for factors potentially associated with antimicrobial resistance in *Campylobacter* species, *Enterococcus* species, *Escherichia coli*, and *Salmonella enterica* from turkeys^a,b^

| **#** | **Searches** | **Results** |
| --- | --- | --- |
| 1 | ANTIBIOTICS RESISTANCE/ | 7440 |
| 2 | ((resistan* adj4 (antimicrobial* or microbial* or antibiotic* or anti biotic* or antibacterial* or bacteria* or multidrug* or multi drug* or extensively drug or multiple drug* or multiclass* or multi class* or multiple class*)) or amr).tw,hw. | 12900 |
| 3^c^ | beta-LACTAM ANTIBIOTICs/ or beta-LACTAMASES/ or exp TETRACYCLINES/ or exp QUINOLONES/ or exp MACROLIDE ANTIBIOTICS/ | 3167 |
| 4^c^ | ("b-lactam*" or "beta-lactam*" or penicillin* or carbapenem* or cephalosporin* or moxalactam* or latamoxef* or tetracycline* or quinolone* or "4-Quinolone*" or fluoroquinolone* or macrolide* or tylosin* or ciprofloxacin* or enrofloxacin*).tw,hw. | 9581 |
| 5 | or/1-4 | 18090 |
| 6 | (exp meat/ or meat products/) and turkeys/ | 1050 |
| 7 | exp turkey meat/ or meleagris gallopavo.tw,hw. | 1887 |
| 8 | (turkeys or turkey or gobbler?).ti,hw. or (turkeys or turkey or gobbler?).ab. /freq=2 | 8035 |
| 9 | or/6-8 | 8039 |
| 10 | ESCHERICHIA COLI/ or (escherichia coli or "e coli" or ecoli).tw,hw. | 41659 |
| 11 | exp SALMONELLA/ or salmonella.tw,hw. | 28842 |
| 12 | exp campylobacter/ or campylobacter.tw,hw. | 6249 |
| 13 | ENTEROCOCCUS FAECIUM/ or (enterococcus faecium or "e faecium").tw,hw. | 1989 |
| 14 | ENTEROCOCCUS FAECALIS/ or (enterococcus faecalis or "e faecalis").tw,hw. | 2029 |
| 15 | or/10-14 | 67445 |
| 16 | and/5,9,15 | 205 |
| 17 | (201904* or 201905* or 201906* or 201907* or 201908* or 201909* or 20191* or 202*).up,ed. | 230173 |
| 18 | 16 and 17 | 37 |
| 19 | (adana or adiyaman or afyonkarahisar or agri or amasya or ankara or antalya or artvin or aydin or balikesir or bilecik or bingol or bitlis or bolu or burdur or bursa or canakkale or cankiri or corum or denizli or diyarbakir or edirne or elazig or erzincan or erzurum or eskisehir or gaziantep or giresun or gumushane or hakkari or hatay or isparta or mersin or istanbul or izmir or kars or kastamonu or kayseri or kirklareli or kirsehir or kocaeli or konya or kutahya or malatya or manisa or kahramanmaras or mardin or mugla or nevsehir or nigde or ordu or rize or sakarya or samsun or siirt or sinop or sivas or tekirdag or tokat or trabzon or tunceli or sanliurfa or usak or yozgat or zonguldak or aksaray or bayburt or karaman or kirikkale or batman or sirnak or bartin or ardahan or igdir or yalova or karabuk or kilis or osmaniye or duzce).tw,hw. | 3587 |
| 20 | (((north* or east* or south* or west*) adj turkey) or turkish).tw,hw. | 2565 |
| 21 | turkey.ad. | 20147 |
| 22 | or/19-21 | 22492 |
| 23 | 18 not 22 | 25 |

^a^The initial search was also altered to filter out terms related to Turkey (the country).

^b^The dates of coverage for this database were 1969 to 2021 August Week 1.

^c^Search terms for extended-spectrum cephalosporins, quinolones, macrolides, and tetracyclines were added to improve the sensitivity of the search for these antimicrobial classes. These classes are of particular relevance to the iAM.AMR project [1].

**Supplementary Table S11**. Description of the qualitative data extraction items for relevant, included references

| **Variable** | **Description** | **Response Type** |
| --- | --- | --- |
| **Reference-level characteristics** |  |  |
| Year of publication | Year of publication | Free text |
| Study location(s) | The country or countries, and, if reported, the sub-region(s) (e.g., province(s), state(s)) where sampling was conducted | Selected from pre-populated responses |
| Study design | Observational, experimental, neither, or was unclear | Selected from pre-populated responses |
| Study design detail | Additional details including, but not limited to, the characteristics of the study population, group allocation of study participants, study facilities (e.g., commercial farms, experimental facilities), blinding of investigators, housing protocols, sampling procedures, data collection surveys or questionnaires | Free text |
| Sampling method | Method of sample selection and collection, including the sample type (e.g., individual samples, barn floor swabs), sample quantity, and the bacterial isolation method (including whether or not antimicrobial selective media was used) | Free text |
| **Factor-level characteristics** |  |  |
| Factor title | Brief description of the reported factor (e.g., ceftiofur use where the potential association between ceftiofur use and antimicrobial resistance was reported) | Free text |
| Factor description | Additional information regarding the factor^a^ and comparator^b^ groups including, but not limited to, information about any antimicrobial use (e.g., dose, duration of use), exposure period, sampling regime, etc. | Free text |
| Factor group^a^ | Short, descriptive title of the group where the factor was present or applied | Free text |
| Comparator group^b^ | Short, descriptive title of the group where the factor was not present or applied | Free text |
| Production stage of factor application | Production stage along the farm-to-fork pathway where the factor was present or applied (e.g., organic production present or applied at the farm) | Selected from pre-populated responses |
| Production stage of factor measurement | Production stage along the farm-to-fork pathway at which the potential association between a factor and antimicrobial resistance was reported (e.g., retail, if a potential association between organic production and antimicrobial resistance was reported for bacteria sampled at retail) | Selected from pre-populated responses |
| Host population | Animal population where the factor was present or applied, and in which antimicrobial resistance was investigated in potential associations with identified factors. This includes sub-populations such as poults, adult turkeys, or turkey carcasses | Selected from pre-populated responses |
| **Outcome-level characteristics** |  |  |
| Bacterial genus or species | The isolated bacteria (genus or species) in which antimicrobial resistance was investigated in potential associations with identified factors | Selected from pre-populated responses |
| Resistance outcome | The specific antimicrobial or antimicrobial class for which antimicrobial resistance was investigated in potential associations with identified factors | Selected from pre-populated responses |
| Unit of analysis | Unit(s) of analysis (e.g., isolate, animal, flock, farm) in which observations of antimicrobial resistance were reported | Selected from pre-populated responses |

^a^Factor group: The study group in which the factor was present or applied (e.g., a group of turkeys in which chlortetracycline was administered in-feed).

^b^Comparator group: The study group in which the factor was not present or applied (e.g., a group of turkeys in which chlortetracycline was not administered in-feed).

**References**

1. **Murphy CP, et al.** (2018) Factors potentially linked with the occurrence of antimicrobial resistance in selected bacteria from cattle, chickens and pigs: a scoping review of publications for use in modelling of antimicrobial resistance (IAM.AMR project). *Zoonoses and Public Health*; **65**: 957–971.
